# Tuning Reconstruction Level of Precatalysts to Design Advanced Oxygen Evolution Electrocatalysts

**DOI:** 10.3390/molecules26185476

**Published:** 2021-09-09

**Authors:** Hainan Sun, Yinlong Zhu, WooChul Jung

**Affiliations:** 1 Department of Materials Science and Engineering, Korea Advanced Institute of Science and Technology, Daejeon 34141, Korea; hainansun0718@kaist.ac.kr; 2 Department of Chemical Engineering, Monash University, Clayton, VIC 3800, Australia; yinlong.zhu@monash.edu

**Keywords:** precatalyst, reconstruction level, surface reconstruction, deep reconstruction, complete reconstruction, OER

## Abstract

Surface reconstruction engineering is an effective strategy to promote the catalytic activities of electrocatalysts, especially for water oxidation. Taking advantage of the physicochemical properties of precatalysts by manipulating their structural self-reconstruction levels provide a promising methodology for achieving suitable catalysts. In this review, we focus on recent advances in research related to the rational control of the process and level of surface transformation ultimately to design advanced oxygen evolution electrocatalysts. We start by discussing the original contributions to surface changes during electrochemical reactions and related factors that can influence the electrocatalytic properties of materials. We then present an overview of current developments and a summary of recently proposed strategies to boost electrochemical performance outcomes by the controlling structural self-reconstruction process. By conveying these insights, processes, general trends, and challenges, this review will further our understanding of surface reconstruction processes and facilitate the development of high-performance electrocatalysts beyond water oxidation.

## 1. Introduction

Energy conversion and storage systems are the most promising solutions to address the energy crisis and environmental pollution [1,2,3,4]. As a part of these solutions, electrically driven water splitting and the metal-air battery are two representative technologies [5,6,7,8]. Due to the complexity of four-electron-proton oxidation, the sluggish kinetics of the OER in electrocatalysis greatly hinders the overall efficiency of these processes [9,10,11,12,13]. Currently, noble metals and their compounds are considered state-of-the-art electrocatalysts for the OER [9,14,15,16,17,18,19,20,21]. To reduce the use of noble metals and ensure better utilization of noble-metal atoms, optimizing emerging design strategies, such as structural engineering, alloying with transition metals, and developing single-atom noble-metal sites are necessary [16,20,21,22]. Meanwhile, noble-metal-based electrocatalysts are greatly limited when used in commercial applications due to their scarcity and high cost. Therefore, tremendous efforts have been devoted to the development of efficient OER catalysts that cost less [17,23,24,25,26,27,28,29,30].

Particularly, first-row transition metal-based materials, such as oxyhydroxides, oxides (e.g., spinel oxides and perovskite oxides), and metal phosphides/chalcogenides reportedly show outstanding OER performance, given their flexible electronic structure and enriched active sites [31,32,33,34,35,36,37,38,39,40,41]. Notably, the electrocatalytic activities of many reported compounds based on nonprecious transition metals are even superior to those of noble-metal-based catalysts [23,42,43,44,45]. To ensure rational designs of precatalysts (catalysts under non-reaction conditions), numerous strategies, such as surface/defect/strain engineering, heteroatom doping, alloying, and the construction of hybrids (e.g., core-shell structure), and nanoparticle-support composites have been widely developed, by which active sites can be precisely modulated [1,24,42,46,47,48,49,50,51,52]. In addition, electro-derived surface reconstruction also plays a significant role in determining electrocatalytic activities [51,53,54,55,56,57,58,59,60]. Advanced in situ/operando techniques have demonstrated that the surfaces of precatalysts tend to be converted partly or completely to oxyhydroxides during the electrochemical oxidation process, acting as actual active species for OER [46,51,61,62,63,64,65]. Although typical in situ-formed species are metal oxyhydroxides for after a reconstruction process, the generated active species can show diverse activities even for the same metal oxyhydroxides, as confirmed by different observed electrocatalytic activities from identical oxyhydroxides on different precatalysts surface [62,66]. This phenomenon further highlights the critical role of the rational design precatalysts.

Furthermore, metal-dissolution-induced reconstruction does not always confer a benefit to electrocatalytic activity. The unintended loss of metal ions from the host lattice often seriously sacrifices the surface crystal configurations to form metal oxyhydroxides with dielectric properties on the catalyst surface, leading to the loss and collapse of the material crystalline matrix [67]. Taking Ba_0.5_Sr_0.5_Co_0.8_Fe_0.2_O_3–δ_ (BSCF) as an example, this is a benchmark OER material for perovskite oxides in alkaline media [68]. A descriptor predicting intrinsic OER reactivity through the *e*_g_-filling of a perovskite oxide was successfully proposed by Shao-Horn et al. and is now widely used by many researchers. However, it is not easy clearly to determine the degree of *e*_g_-filling on the catalyst surface, and most attempts thus far have been measured through bulk-sensitive analysis techniques or predicted only through calculations of the binding energy values of oxygenated species on the catalyst surface [69]. Importantly, surface reconstruction was not fully considered in the design of the OER response descriptor. The same group later reported an amorphous surface (Co(Fe)OOH phase) of BSCF during the OER process, which explained the initially enhanced activity as determined by cyclic voltammetry and potentiostat measurements [70,71]. These works promoted a further understanding of catalyst surface changes under electrochemical potentials, especially by what are known as in situ/operando techniques [50,51,61,72,73,74,75,76]. It is also important to note that further data from electrochemical experiments revealed that BSCF would lose its superior activity after long-term testing, which is more likely due to the collapse of the crystalline matrix on the surface [77]. Our understanding of surface reconstruction has grown progressively and become more refined with the ongoing research. Therefore, a limitation/circumvention strategy and the acceleration of the surface reconstruction process appear to be other effective methodologies that can be used to design high-performance OER electrocatalysts.

**Figure 1:**
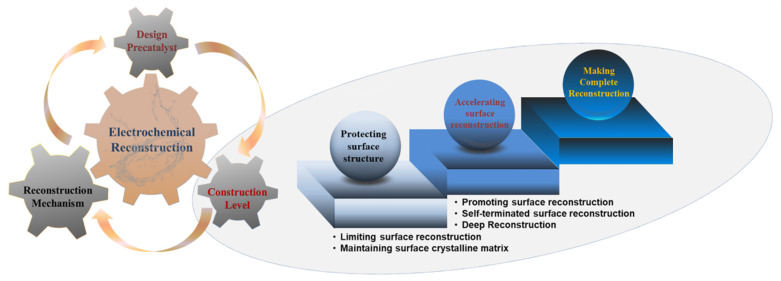
Schematic diagrams of strategies used to control the surface reconstruction process for advanced OER electrocatalysts.

The intent of this review is to summarize recent advances in design strategies for the controllable levels of structural self-reconstruction to develop advanced OER electrocatalysts (Figure 1). We discuss original contributions to the structural self-reconstruction research and the necessity to control surface changes, after which we summarize recently proposed concepts to boost electrocatalytic performance. The challenges and outlooks in this field are also presented in an effort to clarify the current state of surface reconstruction technology and rational designs of high-performance electrocatalysts for the OER.

## 2. Fundamental Understanding of Surface Reconstruction

**Figure 2:**
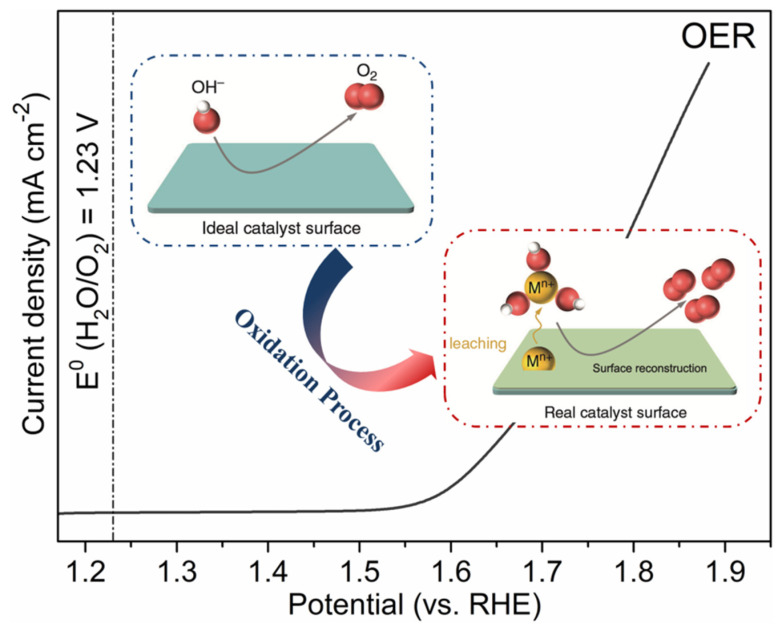
Schematic of the polarization curves of the OER and schematic illustration of the surface reconstruction process. Reproduced with permission [67]. Copyright 2020, Nature Publishing Group.

To gain an in-depth understanding of surface reconstruction coupled with changes in electrocatalytic activity level, it is essential to understand the reaction mechanism of the OER. The equilibrium potential of the OER is 1.23 V vs. a reversible hydrogen electrode at room temperature [26]. The traditionally followed adsorbate evolution mechanism involves different OER intermediates, including HO*, HOO*, and O* species [1,10,65]. The scaling relationships, in which the adsorption energies of different intermediates are linearly correlated, cause the practically applied potential to drive the OER process much higher than the ideal value of 1.23 V [47]. Furthermore, potentials between the applied value in a practical catalytic process and the redox potential of the given catalyst overlap to some extent, which contributes to the occurrence of electrocatalytic activity and catalyst surface structural self-reconstruction at the same time (Figure 2) [53,63,78,79].

The structural properties of in situ-formed species on a catalyst surface mainly determine the final catalytic behavior related to the local electronic structure of the species, such as hydrophilicity, co-ordination, and metal-oxygen covalency [55,80]. Recently, a series of sophisticated in situ/operando characterizations have led to the monitoring of the dynamic structural reconstructions of various types of electrocatalysts [61,63,81,82]. These efforts found that typical in situ-formed species on catalyst surfaces are metal oxyhydroxides [53,62,78,79,82,83,84]. However, the generated surface-active species showed a range of activity even for the same metal oxyhydroxides, a finding also confirmed by the different observed electrocatalytic activities from identical oxyhydroxides on different precatalysts surfaces [62]. These results further highlight the importance of elaborately designing controllable structures of precatalysts. In addition, factors that affect the reaction conditions such as the concentration of the electrolyte, the applied potential, and the temperature will also affect the degree of surface change [56,85]. Thus, much effort has been devoted to exploring advanced OER electrocatalysts based on surface reconstruction [63,79,86,87].

## 3. Necessity to Control Surface Reconstruction

Based on the above-mentioned descriptions, surface reconstruction is an ubiquitous electrochemical phenomenon during the OER process [78,79]. However, surface reconstruction engineering is a comprehensive and complex process, which requires the rational design of the precatalyst and precise control of the surface change process [7,63,88]. The parameters of phase/electronic/geometrical structures, oxidation/spin states, and the morphology should be systemically considered [53,79,89,90,91,92,93]. Furthermore, in situ/operando spectroscopic techniques are crucial to identify the actual active species, providing greater insights into the relationship between the structure and activity of the studied catalysts [45,75,82,87,94,95,96,97,98].

Therefore, how rationally to control the surface change, including the rate and level of surface reconstruction, during the OER process to design high-performance OER electrocatalysts is an open question. Herein, to demonstrate the feasibility of this concept, current developments, and recently proposed strategies to boost electrocatalytic performance capabilities by limiting/circumventing/accelerating surface reconstruction processes are discussed below.

## 4. Protecting Surface Structure

### 4.1. Limiting Surface Reconstruction

**Figure 3:**
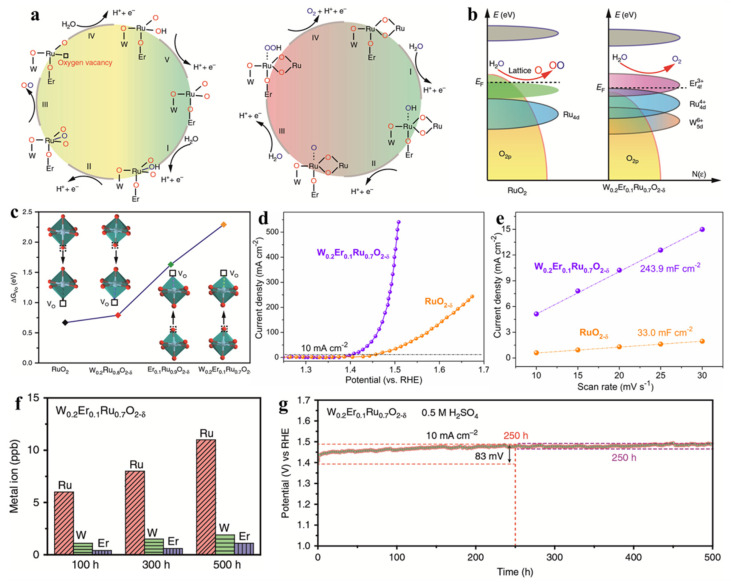
(**a**) OER reaction mechanisms of LOM and AEM for RuO_2_ in acidic media. (**b**) Schematic diagrams of the band model for RuO_2_ and co-doped RuO_2_ for the OER in acidic media. (**c**) Calculated oxygen vacancy formation energy at different positions of RuO_2_, single-doped RuO_2_, and co-doped RuO_2_. (**d**) LSV curves of RuO_2_ and co-doped RuO_2_ for the OER in a 0.5 M H_2_SO_4_ solution. (**e**) Linear fitting of the capacitive current densities vs. CV scan rates for RuO_2_ and co-doped RuO_2_. (**f**) Metal ion dissolution during the OER process. (**g**) Stability tests for the co-doped RuO_2_ catalyst. Reproduced with permission. [102] Copyright 2020, Nature Publishing Group.

Due to the moderate capacity of binding oxygen, noble-metal oxide RuO_2_ is considered as a benchmark OER electrocatalyst, especially in acidic media [9,21,99]. However, the operational stability of RuO_2_ is a serious issue to be overcome [90,99]. Great efforts have been devoted to improving the stability of RuO_2_ through various types of strategies, such as introduction of heterogeneous elements, strain engineering, and construction of strongly coupled RuO_2_/non-noble-metal hybrids [20,90,99,100,101]. The deterioration mechanism of RuO_2_ is ascribed to the over-oxidation of Ru due to the oxidation of lattice oxygen. The generation of oxygen vacancies caused Ru atoms to be exposed on the catalyst surface, which are then further oxidized to soluble materials (e.g., high-valence-state material RuO_4_) (Figure 3a) [9,102]. Surface reconstruction at the expense of the surface crystal structure harms the operational stability [102]. Therefore, it would be reasonable to mitigate this unfavorable surface change if the formation energy of oxygen vacancies is much greater than the redox H_2_O/O_2_ energy. Based on this design concept, Hao et al. utilized a foreign doping strategy to enlarge the localized gap between O *2p* band centers and the Fermi level, which can enhance the energy barrier for lattice oxygen oxidation and effectively prohibit oxygen vacancies (Figure 3b,c) [102]. The introduction of Er and W into the lattice of RuO_2_ modulates its electronic structure. The density of states from density functional theory (DFT) calculations suggests that the gap between O *2p* band centers and the Fermi level was enlarged from −3.31 eV (RuO_2_) to −4.12 eV (W_m_Er_n_Ru_1−m−n_O_2−δ_), indicating that the covalency of the Ru-O bond was decreased by the co-doped effect. Thus, the reaction mechanism of the W_0.2_Er_0.1_Ru_0.7_O_2−δ_ catalyst in an acidic solution would tend to follow the adsorbate evolution mechanism rather than the lattice oxidation mechanism because it is thermodynamically unfavorable for direct O-O coupling of O *2p* states with regard to the Fermi level (Figure 3a). In addition to the obviously enhanced activity, in terms of the overpotential and current densities (Figure 3d), the co-doped effect can also lead to a considerable number of active sites exposed on the catalyst surface (Figure 3e). Moreover, the dissolution of metal cations (especially Ru cations) would be successfully suppressed, as revealed by the relatively low and stable concentrations of the dissolved cations in the solution (Figure 3f). The W_0.2_Er_0.1_Ru_0.7_O_2−δ_ catalyst could survive in highly oxidative and corrosive conditions for at least 350 h, highlighting its superior operational stability.

### 4.2. Maintaining Surface Crystalline Matrix

The OER reaction is an oxidation process, during which low-valence metal sites tend to be oxidized to higher valence states along with metal dissolution into the electrolyte [17,53,92,93,98]. Following this, surface reconstruction occurs at the expense of the loss and collapse of the surface crystalline matrix with the formation of an amorphous (oxy)hydroxide layer on the surface [103,104,105]. In contrast, transition-metal ions with high valence states (e.g., Co^3+^/Co^4+^ couples and Fe^4+^ ions) can relieve the electro-derived oxidation process and exhibit good structural/operational stability levels, as demonstrated with recently reported high-performance oxides, such as SrCoO_3_, Na_0.67_CoO_2_, Ba_4_Sr_4_(Co_0.8_Fe_0.2_)_4_O_15_, and CaCu_3_Fe_4_O_12_ [26,106,107,108,109]. In addition, regarding perovskite oxides, the leaching of ions (cation leaching and anion leaching) is inevitable during the OER process in both alkaline and acidic media [17,18,79,104,110,111,112].

**Figure 4:**
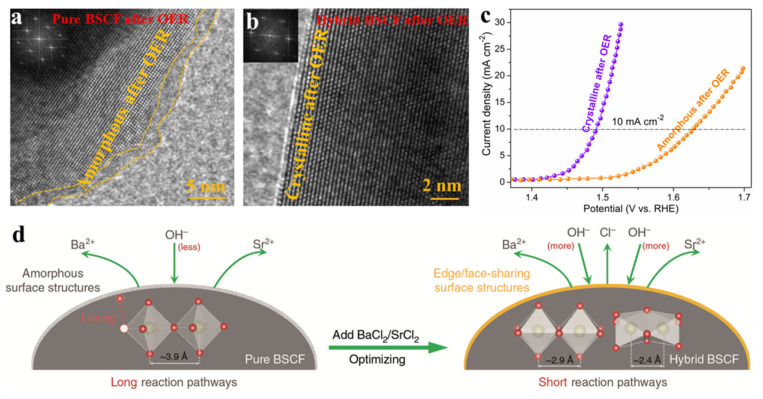
HRTEM images of the (**a**) pure-phase BSCF and (**b**) hybrid BSCF after OER tests. (**c**) LSV curves of the pure-phase BSCF and hybrid BSCF for the OER. (**d**) Schematic illustrations showing control of the surface change. Reproduced with permission [67]. Copyright 2020, Nature Publishing Group.

Considering these factors, Guan et al. proposed a novel design principle by which to optimize the surface reconstruction process for perovskite oxides. In detail, soluble materials (e.g., SrCl_2_ and BaCl_2_) were introduced into the perovskite structure with high-valence-state transition-metal ions simultaneously to achieve an optimized cation/anion leaching effect and to keep the surface crystalline matrix unchanged during the harsh reaction conditions [67]. During the reaction process, soluble materials leached into the electrolyte, as confirmed by the nearly absent Cl 2p XPS spectra, leading to a balance of the alkaline-earth metal ion concentration between the host phase and the interfacial liquid. Benefitting from this unique leaching effect, the perovskite surface crystalline matrix was well preserved after the harsh oxidation conditions compared to that of the pure perovskite phase (Figure 4a,b). As a result, a perovskite with a well-preserved crystalline surface after the OER exhibited dramatically improved OER activity compared to that of the amorphous sample. Impressively, the hybrid catalyst requires a low overpotential of 260 mV to obtain a current density of 10 mA cm^−2^, which is approximately 137 mV lower than that of the pure-phase material (Figure 4c). Notably, the OER activity of the hybrid catalyst represents the top level among recently reported perovskite oxide materials. Thus, the proposed design strategy can effectively change the reaction mechanism, allowing control of the surface reconstruction process (Figure 4d). In addition, the proposed methodology is a universal method that can also be applied to many types of oxides, such as single perovskites, double perovskites, and the Ruddlesden–Popper perovskite.

## 5. Accelerating Surface Reconstruction

### 5.1. Promoting Surface Reconstruction

The controllable leaching can promote the rate and level of surface reconstruction, which effectively increases the number of active sites on the catalyst surface. Moreover, the rate of anion/cation leaching is also dependent on the applied anodic potential. In this section, we briefly summarize the effects of cation and anion leaching for promoting surface reconstruction during the OER process.

**Figure 5:**
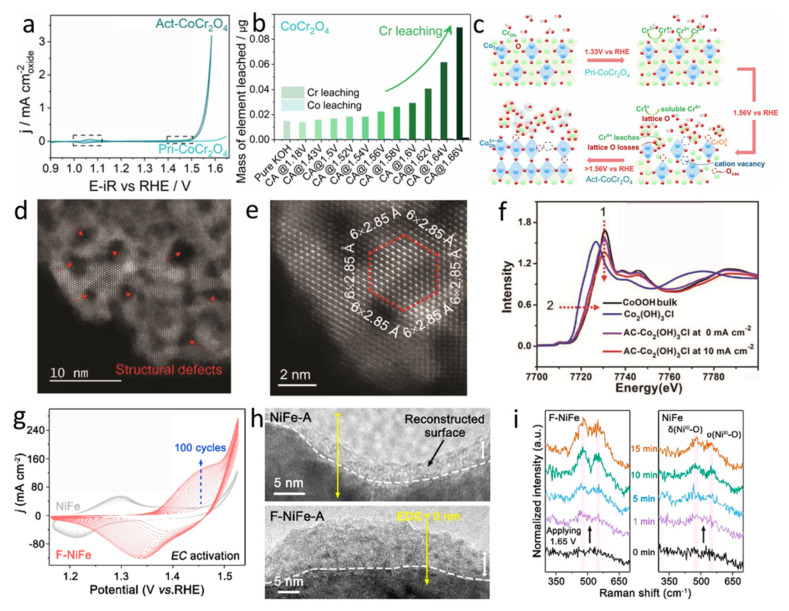
(**a**) CV curves of CoCr_2_O_4_ with the initial state (2nd cycle) and after chronoamperometry (at 1.7 V for 1.5 h). (**b**) The cumulative mass of Co and Cr leached after consecutive series of chronoamperometry conducted at 20mins interval for CoCr_2_O_4_. (**c**) Scheme of CoCr_2_O_4_ surface reconstruction before OER testing after being applied increased potentials. Reproduced with permission [113]. Copyright 2021, Wiley-VCH. (**d**) STEM and (**e**) atomic-resolution HAADF-STEM images of the AC-Co_2_(OH)_3_Cl. (**f**) Normalized Co K-edge XANES spectra of the Co_2_(OH)_3_Cl, AC-Co_2_(OH)_3_Cl under OER and non-OER conditions as well as the reference (CoOOH bulk). Reproduced with permission [66]. Copyright 2019, Wiley-VCH. (**g**) CV curves of the F-NiFe and NiFe in an alkaline solution. (**h**) HRTEM images of F-NiFe-A and the NiFe-A. (**i**) Operando Raman spectra of F-NiFe and the NiFe. Reproduced with permission [114]. Copyright 2021, American Chemical Society.

Duan et al. reported controllable anodic leaching of Cr in a spinel oxide CoCr_2_O_4_ to obtain outstanding OER activity (Figure 5a) [113]. Remarkably, the higher the potential is applied the high concentration of Cr in the solution is detected (Figure 5b). In contrast, the level of Co leaching is independent of the applied potentials (Figure 5b). Moreover, the levels of Cr leaching are consistent with the increased electrocatalytic activities. First, the vacancies and defects induced by Cr leaching enable the formation of Co oxyhydroxide. Second, under higher anodic potentials, the further promoted Cr leaching and lattice oxygen consumption make reconstructed Co species with higher intrinsic activity per site (Figure 5c). Song’s group reported cobalt oxychloride Co_2_(OH)_3_Cl that exhibited a gradual phase transition due to the etching of lattice Cl^−^ ions, alone with continuously increased OER activities [66]. The activated material shows an overpotential of 270 mV at 10 mA cm^−2^ in an alkaline solution, which is comparable to that of noble metal oxide IrO_2_. The irreversible etching of the lattice Cl^−^ brought about numerous structural defects and high-valence Co species, which are demonstrated by aberration-corrected high-angle annular dark-field scanning transmission electron microscopy and operando synchrotron radiation-based X-ray spectroscopic characterizations, respectively (Figure 5d–f). Moreover, first-row transition metal sulfides, phosphides, and nitrides are also promising candidates for OER electrocatalysts in an alkaline environment. Of note, the surfaces of these materials are more prone to be oxidized to metal oxyhydroxide due to the fast surface reconstruction under the electrochemical oxidation condition [57,84,114]. Xu et al. evaluated the effect of the introduction of F into NiFe structure, which can enable a faster and deeper surface reconstruction process (Figure 5g) [114]. In detail, the precatalyst of NiFeO_x_F_y_ nanosheets are easily changed into Ni(Fe)O_x_H_y_ compared with the initial NiFe precatalysts, which was demonstrated by operando Raman, ex-situ XPS spectra, and HRTEM analyses (Figure 5h,i). The positive effect of F-modification is also demonstrated in metal sulfide materials. For example, the surface of fluorinated Ni_3_S_2_ exhibits a low-crystalline nanosheet structure with obvious Ni-F bonds, which can greatly contribute to the formation of active NiOOH active species [115]. Additionally, the advantages of good conductivity and fast reaction kinetics of the fluorinated Ni_3_S_2_ catalyst jointly bring about the enhanced OER performance.

### 5.2. Realizing Self-Terminated Surface Reconstruction

Inactive and low-cost materials as potential catalysts should be activated as high-performance OER electrocatalysts. In situ-generated active species (e.g., Co^3+^ oxyhydroxides) after surface reconstruction can serve as main active sites, offering high activity capabilities [55,62,86,116]. The challenge is how rationally and precisely to initiate the surface reconstruction of inactive catalysts. Furthermore, the restructuring degree should be kept under control. In many reported cases, bulk precatalysts serving as a template would be partly (or even completely) compromised to create a highly active surface. Therefore, the development of strategies for activating and terminating the surface reconstruction process is crucial.

**Figure 6:**
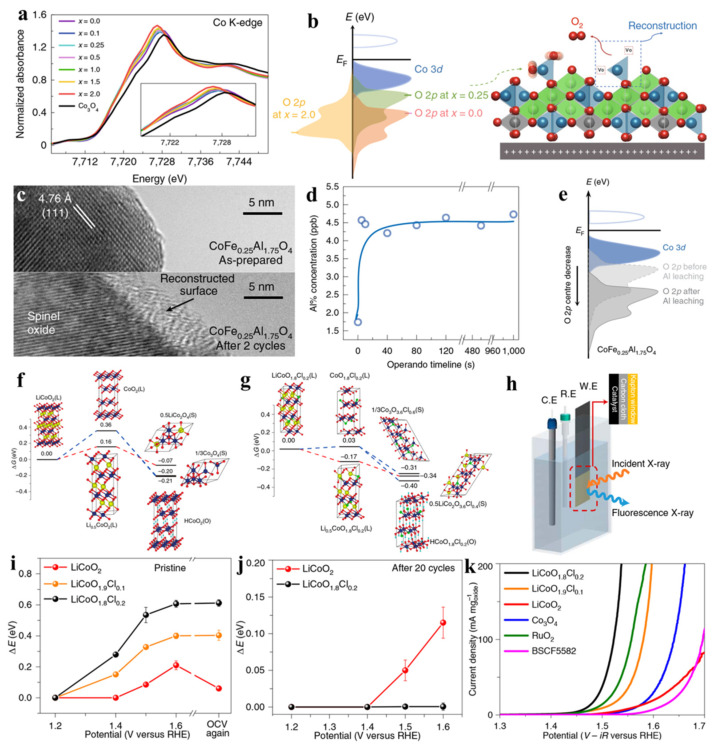
(**a**) Co *K*-edge XANES spectra of Fe-doped CoAl_2_O_4_ samples. (**b**) Schematic band illustrations of CoAl_2_O_4_, CoFe_0.25_Al_1.75_O_4_, and CoFe_2_O_4_, and schematic demonstration of the surface reconstruction of CoFe_0.25_Al_1.75_O_4_. (**c**) HRTEM images of the CoFe_0.25_Al_1.75_O_4_ catalyst before and after OER tests. (**d**) Al concentration in the reaction electrolyte during the OER process. (**e**) Comparison of the band structure of CoFe_0.25_Al_1.75_O_4_ with and without Al^3+^ vacancies. Reproduced with permission [104]. Copyright 2019, Nature Publishing Group. Reaction energy profiles at 1.6 V versus RHE for (**f**) LiCoO_2_ and (**g**) LiCoO_1.8_Cl_0.2_. (**h**) Experimental set-up scheme for operando testing. (**i**) Co K-edge shift at different potentials for fresh samples. (**j**) Co K-edge shift for cycled samples. (**k**) OER mass activity for LiCoO_2_, LiCoO_1.9_Cl_0.1_, LiCoO_1.8_Cl_0.2_, Co_3_O_4_, BSCF, and RuO_2_. Reproduced with permission [73]. Copyright 2021, Nature Publishing Group.

Wu et al. reported Fe-doped CoAl_2_O_4_ to showcase this design strategy in the development of high-performance OER electrocatalysts based on inactive and low-cost spinel oxides [104]. After Fe substitution, a decrease in the number of Co valence states with the creation of oxygen vacancies and a raised O 2p level were observed for a CoFe_0.25_Al_1.75_O_4_ sample, as confirmed by X-ray absorption near-edge structure (XANES) spectroscopy (Figure 6a,b). Modulation of the pre-oxidation states of Co^2+^ in CoFe_0.25_Al_1.75_O_4_ provides a great potential for surface reconstruction to form surface-active species. In addition, an irreversible surface change with the generation of surface oxyhydroxide was demonstrated by the electrochemical behavior, with negligible changes of the pseudocapacitive charge after the second cyclic voltammetry (CV) cycle. For perovskite oxides, if lattice oxygen participates in the reaction, a descriptor of the O *2p* level relative to the Fermi level can be involved during the reaction process. However, with regard to CoFe_0.25_Al_1.75_O_4_, a ≈5 nm reconstructed surface was found after the first cycle, remaining quite stable during subsequent cycling (Figure 6c) and revealing the unlikely involvement of lattice oxygen during the reconstruction process. That is to say, surface reconstruction was triggered by lattice oxygen oxidation and could be quickly terminated after the first cycle. The reconstruction process was closely related to the leaching of metal cations, especially for the Al metal ions. The leaching of Al cations was found to be quite remarkable compared to that of Co and Fe cations. Importantly, Al leaching would end quickly, revealing a stable concentration of Al in the tested electrolytes stemming from the initial reaction process (Figure 6d). Furthermore, DFT calculations revealed that the energy of the O *2p* level decreased when Al vacancies were introduced into the lattice (Figure 6e). Similar to the W_0.2_Er_0.1_Ru_0.7_O_2−δ_ case, the lattice oxygen oxidation would end when the energy of the O *2p* level was low. Thus, the reconstruction process stopped as no additional oxygen vacancies were created. Benefitting from the above-mentioned advantages, the cost-effective CoFe_0.25_Al_1.75_O_4_ catalyst exhibited superior mass activity compared to that of the noble-metal oxide IrO_2_ and benchmark transition-metal oxides (e.g., BSCF and Pr_0.5_Ba_0.5_CoO_3−δ_).

The proposed surface reconstruction self-terminated methodology can also be applied to other types of materials. Very recently, Wang et al. redirected the dynamic surface restructuring of layered transition-metal oxides [73]. From the DFT-calculated reaction energy profile, delithiation is energetically unfavorable for the LiCoO_2_ oxide and requires a higher potential level to trigger surface reconstruction to Li_1±x_Co_2_O_4_ and longer cycles for stabilization (Figure 6f). Cl-doping can reduce the electrochemical potential to initiate cobalt oxidation and lithium leaching, causing to the surface to transform into a self-terminated amorphous (oxy)hydroxide (Figure 6g). The operando XAFS spectra at the Co K-edge were collected to uncover the role of doped-Cl (Figure 6h). A decrease in the initial Co valence state of LiCoO_1.8_Cl_0.2_ leads to a greater increase in the Co valence than that in LiCoO_2_ within the same anodic potential range (Figure 6i). Notably, the restructuring process for LiCoO_1.8_Cl_0.2_ is completely irreversible, as demonstrated by the negligible change of the XANES outcomes after 20 CV cycles. In contrast, for the cycled sample of LiCoO_2_, the corresponding Co K-edge shifted further during the electrochemical reaction (Figure 6j), indicating that either the restructuring process was not complete or that the restructured phase was unstable. Moreover, transition-metal oxyhydroxides are reportedly more active than their spinel counterparts. The OER activity of surface-restructured LiCoO_1.8_Cl_0.2_ outperformed many state-of-the-art OER catalysts. By manipulating the in situ catalyst leaching, this work deepens our understanding of modulated surface restructuring (Figure 6k).

### 5.3. Realizing Deep Reconstruction

Independent of material types and particle sizes, for most reported OER-precatalysts, the values of reconstruction layer thickness are less than 10 nm [85]. Considering the highly active of in situ formed metal oxyhydroxides, it is reasonable to expect that the electrochemical performance of precatalysts would be greatly improved with the enhanced surface reconstruction level, namely deep surface reconstruction [117,118]. Limited by the characteristic of the electrochemical process (the catalysis takes place on the surface of catalysts) and catalyst structures (e.g., particle size and morphology), it is still changing to realize the deep reconstruction and the related universal design methods are strongly demanded.

**Figure 7:**
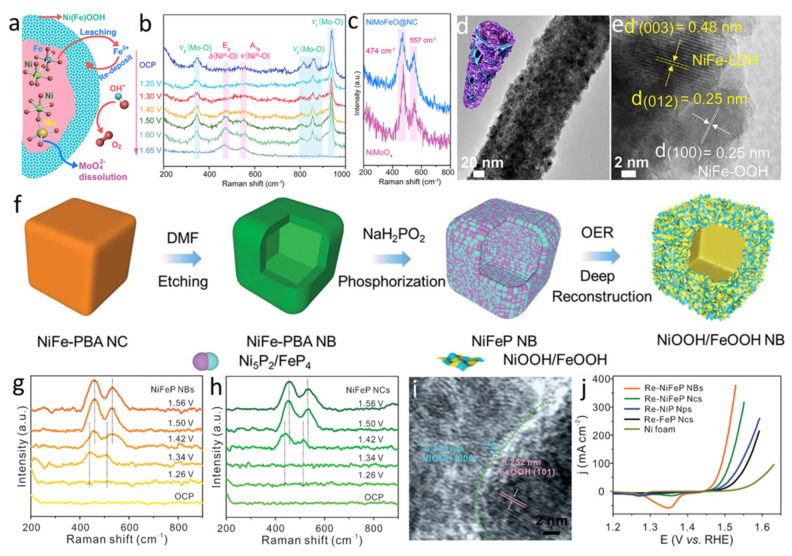
(**a**) Schematic of the reconstruction process for NiMoFeO@NC. (**b**) Operando Raman spectra of NiMoFeO@NC. (**c**) Operando Raman spectra of NiMoFeO@NC and NiMoO_4_ obtained at 1.65 V. (**d**) Low-magnification and (**e**) high-magnification TEM images of NiMoFeO@NC after electrochemical reaction. Reproduced with permission [119]. Copyright 2020, Cell Press. (**f**) Schematic for the formation of precatalysts and deep reconstruction process of precatalysts into hierarchical NiOOH/FeOOH structures. Operando Raman spectra of (**g**) NiFeP NBs and (**h**) NiFeP NCs at different applied potentials. (**i**) HRTEM image of NiOOH/FeOOH structures. (**j**) LSV curves of catalysts loaded on Ni foam after 30 h of reconstruction process. Reproduced with permission [118]. Copyright 2021, Royal Society of Chemistry.

Wang et al. reported that a rapid and deep self-reconstruction of precatalysts could be realized by an anion etching process (Figure 7a) [119]. The precatalyst is made of a core-shell structure, where NiMoO_4_ is the core and NiFe/Ni-FeO_x_ nanoparticles in N-doped amorphous carbon is the shell. The fast dissolution rate of MoO_4_^2−^ and incorporation of Fe in the Ni-oxyhydroxide leads to the in situ generation of NiFeOOH/NiFe LDH (Figure 7b–e), which contributes to the rapid and deep self-reconstruction process. Yan et al. utilized the advantage of hollow structure to promote the reconstruction level (Figure 7f) [118]. In particular, the in situ Raman spectroscopy confirmed the positive effect of hollow structure on facilitating the deep reconstruction (Figure 7g,h). In detail, the precatalyst of Ni_5_P_2_/FeP_4_ nanoboxes deeply reconstruct into NiOOH/FeOOH nanosheet (Figure 7i). Moreover, the considerable interface between FeOOH and NiOOH and abundant defects jointly contribute to superior OER activity (Figure 7j).

## 6. Making Complete Reconstruction

For surface reconstruction and deep reconstruction, the in situ formed layer would lead to a core-shell structure, where the in situ-formed active species and the unchanged precatalyst serve as the shell and core, respectively [14,63,75,92,104,120,121,122]. When the core material cannot favorably contribute to the shell or when the core material has the potential to be transformed further into active species, traditional near-surface reconstruction would have considerable limitations [85]. Complete changes of bulk materials can enhance the active sites to a great extent, but this remains a major challenge [85,123].

**Figure 8:**
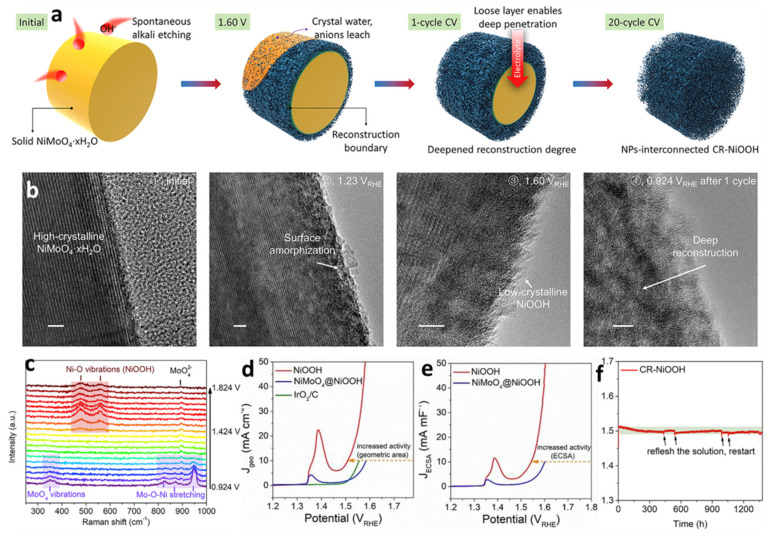
(**a**) Schematic diagrams of the complete surface change process for NiMoO_4_**·**xH_2_O. (**b**) Ex situ HRTEM images of the different reconstruction degrees at various activated stages of NiMoO_4_·xH_2_O. Scale bars, 5 nm. (**c**) In situ Raman spectra of NiMoO_4_·xH_2_O during the OER process. LSV curves of the studied catalysts for the OER normalized by the (**d**) geometric area and (**e**) electrochemically active surface area. (**f**) Ultra-long stability test of the completely reconstructed NiOOH at a current density of 10 mA cm^−2^. Reproduced with permission [85]. Copyright 2020, Cell Press.

Very recently, Liu and his-worker demonstrated a complete-change mechanism [85]. The key factor to induce complete reconstruction is to construct a loose surface layer. For the NiMoO_4_ catalyst, a low degree of surface reconstruction was found due to a dense restructured layer, leading to a core-shell NiMoO_4_@NiOOH structure. The strategy of etching–leaching engineering was presented to realize the complete reconstruction effect in a bulk hydrate precatalysts system. Specifically, for the rational designed precatalyst of NiMoO_4_·xH_2_O, the co-leaching of soluble Mo species and crystal water during the OER process in an alkaline solution created a considerable nanoporous structure with the generation of high-valence Ni^3+^ species (NiOOH). Finally, the generated nanoporous and loose layer played a crucial role in triggering the full diffusion of electrolytes into the inner structures, by which the goal of a complete change was achieved (Figure 8a). The surface change process involved microstructures of intermediates, as was visually revealed in ex situ HRTEM images (Figure 8b). The process consisted of an amorphous surface at an ultralow overpotential, an additional change at a high overpotential (including NiOOH, amorphous area, and NiMoO_4_·xH_2_O), and complete reconstruction. The potential-dependent in situ Raman spectra further demonstrated the reaction mechanism. Bond breakage, a co-leaching effect, and active species generation could be confirmed by the disappearance of peaks (at ≈ 355 cm^−1^ corresponding to MoO_4_ vibration and at ≈820 and 950 cm^−1^ corresponding to Mo-O-Ni stretching in NiMoO_4_·xH_2_O) and the generation of new peaks with relatively high intensity levels (at 474 and 554 cm^−1^ corresponding to the Ni-O vibration of NiOOH and 900 cm^−1^ corresponding to MoO_4_^2−^ in an alkaline solution) (Figure 8c). The superior electrocatalytic activities of the complete reconstructed catalyst were demonstrated by LSV curves normalized to a geometric area (Figure 8d) and an electrochemically active surface area (Figure 8e). The completely reconstructed catalyst (NiOOH) exhibited clearly enhanced activity compared to the surface-reconstructed catalyst (NiMoO_4_@NiOOH with a core-shell structure) and a commercial noble-metal-based Ir/C catalyst. More importantly, a negligible change of the potential during the 1350 h stability test according to chronopotentiometric measurements was observed for the completely reconstructed catalyst, suggesting vast superiority of the in situ-formed microstructure by the complete reconstruction and also excellent potential as a cost-effective alternative to noble-metal oxides (Figure 8f).

## 7. Conclusions and Outlooks

Rational control of the surface reconstruction process is an effective strategy to develop high-performance OER electrocatalysts (Table 1). This calls for the overall consideration of rationally design precatalysts, an in-depth understanding of the surface reconstruction mechanism, and the exploration of effective design strategies (Figure 1). In this review, recent progress and design strategies related to this topic are summarized for advanced OER electrocatalysts. Beyond these processes to date, there remain many challenges and opportunities for those involved in the design of next-generation catalysts.

### 7.1. Rational Design of Precatalysts

#### 7.1.1. Design Precatalysts

The most widely reported active layers after surface reconstruction are oxyhydroxides [62]. Thus, the relationship between the restructuring behavior and the observed electrocatalytic activity is a significant research topic for those involved in the design of OER catalysts. Additionally, for most catalysts after surface-structure self-reconstruction, a core-shell structure is formed. The in situ generated active layer and the remaining precatalyst act as the shell and the core, respectively [24,87]. Thus, the requirements of the core located between the current collector and the shell structure are also critical.

#### 7.1.2. Reaction Mechanisms

Though in situ-formed species during structural self-reconstruction work as active sites for the OER, the reconstruction mechanism is closely related to the physicochemical properties of the precatalysts used. For example, unlike the conventionally reported irreversible surface change, Bergmann found the structurally reversible change of the crystalline Co_3_O_4_ catalyst [124]. In contrast to the crystalline Co_3_O_4_ catalyst, when an oxygen vacancy was introduced into Co_3_O_4_, the oxygen vacancy could initialize the surface reconstruction process at a relatively low potential, even before the occurrence of the OER process, compared with that of the pure spinel Co_3_O_4_ catalyst [125]. Therefore, rational design of the precatalyst is the key factor determining the electrocatalytic activities and reaction mechanism. Exploring innovative strategies to design high-performance electrocatalysts is of great importance. In addition, if the proposed design strategy is universal, it can guide the design of more advanced electrocatalysts.

#### 7.1.3. New Catalyst Design Concepts

The dissolution of metals (e.g., alkaline and alkaline earth elements) into electrolytes to induce surface reconstruction is widely reported in relation to the design of high-performance electrocatalysts [14,89,110,126,127,128]. The observed electrocatalytic process is highly dependent on the dissolution rate and the final surface species, which form; this is also related to the applied potential and time under an anodic condition [84,128,129]. Thus, the continuous dissolution may be a drawback in terms of long-term application. For example, currently reported OER materials can only work a few hours at a low current density (e.g., 10 mA cm^−2^ is typically used) [130,131]. For practical applications, a high current density, high potential, and high gas production rate are often involved; the shedding and dissolution of the catalysts are tough problems to solve [23,130,132,133]. To avoid these intractable issues, very recently the Cao group proposed a novel concept similar to homogeneous catalysis to fabricate in situ regenerative electrodes that could exhibit high OER activity and superior long-time stability level when operated at a high current density [129]. Specifically, precatalysts were demonstrated as the Co and Fe bimetal salt solutions, which were mixed with an alkaline solution before the reaction. Notably, a conventional three-electrode set-up was used, with bare carbon paper acting as the working electrode initially. During the electrochemical process, homogeneous Co and Fe ions in the electrolyte were consumed and in situ transformed into active species on the surface of the carbon paper at the applied OER potentials. As indicated by the OER curves, the OER activities were gradually enhanced with an increase in the number of CV cycles, which was also confirmed according to the increased active species tested by the electrochemically active surface area. The in situ generation process of active sites could reach a steady state, as reflected by the unchanged activity after 400 CV cycles. Systematical characterizations were conducted to the study changes of the active species after they were formed on the surface of the carbon paper. These results showed that the initial CoFe hydroxides would be in situ transformed into CoFe oxyhydroxide on the substrate surface during the oxidation process. Moreover, in contrast to ex situ techniques, in situ XANES provides fine-grained information about the electronic and geometric structures. For example, the in situ Co XANES result demonstrated that Co^3+^ ions could further be oxidized to Co^4+^ ions, as confirmed by a positive energy shift and a shorter Co-O bond compared with that of the ex situ Co XANES. This work provides a novel strategy to design highly active and stability electrodes.

### 7.2. In-Depth Understanding of Reconstruction Mechanisms

Depending on the types of materials used (e.g., spinel oxide, perovskite oxide, layered oxide and metal oxide-based composites) and the different physicochemical properties of the precatalysts, the actual surface reconstruction mechanism can differ [9,63,79,97]. For example, the surface structure of the perovskite oxide LaCoO_3_ was reported to be quite stable under cycling conditions [62,69,134]. Based on the available literature, stable OER performance under continuous cycling demonstrate there is likely no or negligible surface changes. Regarding the above-mentioned CoFe_0.25_Al_1.75_O_4_ catalyst, there was a quick self-termination process during the OER, [104] while the thickness of the reconstructed surface layers depends strongly on the number of OER cycles for most reported catalysts (e.g., La_2_CoMnO_6_ and SrIrO_3_ thin films) [60,103]. Furthermore, Zhou et al. reported another reconstruction mechanism by which the valence state of a transition metal (Co) under OER conditions was highly dependent on the applied voltage and reaction times, whereas the spin state of Co ions and the edge-sharing Co-O network remained unchanged [93]. Therefore, to gain a deeper understanding of the reaction mechanisms for these types of designed catalysts, comprehensive studies are needed. More importantly, recently developed descriptors to predict the electrochemical activity level of designed catalysts are generally based on the physicochemical properties of the precatalysts without consideration of dynamic changes during the electrochemical reaction [49,135,136,137]. Therefore, developing activity descriptors based on the physicochemical properties of precatalysts and on the surface reconstruction process remains a challenging task that is also urgently needed.

### 7.3. In Situ/Operando Techniques

Generally, it is difficult to capture intermediates with a short lifetime, or to observe dynamic changes by ex situ characterization approaches. To track the surface change during an electrochemical reaction, in situ/operando techniques, specifically in situ/operando XRD/XPS/TEM/XAS/Raman methods, are the most powerful tools [61,63,82,94,138,139]. For example, in situ XAS can provide informative data regarding dynamic changes of the valence state, coordination number, and bond length [72,81,94,140]. Meanwhile, the in situ/operando Raman technique is sensitive to structural changes at different stages under a wide range of electrochemical reaction conditions [72,82,97,141]. It is important to note that it is also difficult to obtain an in-depth understanding of the reconstruction mechanism by only a single in situ/operando technique because each technique has a distinct role due to its application scope [82]. Thus, to reveal precisely the reaction mechanism pertaining to surface reconstruction, a combination of various techniques is needed [63,67,142]. For example, a representative work by Chen’s group utilized three in situ techniques to unravel the real-time changes of a state-of-the-art bifunctional catalyst [143]. Specifically, to determine the dynamic changes that affect the morphology, interface state, and chemical state, in situ TEM, in situ Raman, and in situ XAS were utilized, respectively.

### 7.4. Bridging the Gap between Fundamental Designs and Practical Applications

Considerable efforts have been devoted toward designing high-performance electrocatalysts in fundamental research. Typically, activity evaluations on the laboratory scale are conducted at room temperature [10]. However, there may be a gap between fundamental designs and practical applications. For example, the industrial process of alkaline water electrolysis is operated at 50–80 °C in a high concentration of electrolyte (≈30 wt.% KOH) [56,85,144]. Differences in the surface reconstruction mechanisms between room temperature and industrial temperatures were studied by Liu et al [56]. A thermally induced complete reconstruction phenomenon was found, which is much different from that of catalysts, which operate at room temperature. Therefore, proper control of the surface reconstruction process under practical application conditions is an ongoing issue.

## Figures and Tables

**Table 1:** Summary of OER performance of discussed catalysts in the main text.

Catalysts	Reconstruction Strategies	Overpotential (mV)	Tafel Slope (mV dec^−^^1^)	Stability	Ref.
W_0.2_Er_0.1_Ru_0.7_O_2−δ_	Limiting surface reconstruction	168 @ η10	66.8	η10 @ 500 h	[102]
hybrid BSCF	Maintaining surface crystalline matrix	260 @ η10	N.A.	10 mA @ 100 h	[67]
Co_2_(OH)_3_Cl	Promoting surface reconstruction	270 @ η10	155	η10 @ 10 h	[66]
NiFeO_x_F_y_	Promoting surface reconstruction	218 @ η10	31	η10-200 @ 50 h	[114]
NiFe-OH-F-SR	Promoting surface reconstruction	228 @ η100	22.6	η50 @ 60 h	[66]
F-Ni_3_S_2_	Promoting surface reconstruction	239 @ η10	36	η50 @ 24 h	[115]
CoFe_0.25_Al_1.75_O_4_	Self-terminated surface reconstruction	~270 @ η~3.5	N.A.	η~3.5 @ 48 h	[104]
LiCoO_1.8_Cl_0.2_	Self-terminated surface reconstruction	270 @ η10	55.4	η20 @ 500 h	[73]
Self-Reconcat NiMoFeO@NC	Deep reconstruction	270 @ η50	66.6	η100 @ 24 h	[119]
Ni_5_P_2_/FeP_4_	Deep reconstruction	246 @ η10	41	η50 @ 50 h	[118]
NiMoO_4_·xH_2_O nanowires	Complete reconstruction	278.2 @ η10	N.A.	η10 @ 1350 h	[85]

## Data Availability

Not applicable.
